# Reliability and validity of the PROMIS-29 health profile in Ankylosing Spondylitis patients: A cross-sectional study

**DOI:** 10.1097/MD.0000000000037251

**Published:** 2024-03-01

**Authors:** Mark C. Hwang, Cynthia Bell, Yvette Farran, Alexis Ogdie, Charles Green, John Reveille

**Affiliations:** a Division of Rheumatology, Department of Internal Medicine, John P. and Kathrine G. McGovern School of Medicine, The University of Texas Health Science Center at Houston, Houston, TX, USA; b Center for Clinical Research & Evidence-Based Medicine, Department of Pediatrics, John P. and Kathrine G. McGovern School of Medicine at The University of Texas Health Science Center at Houston, Houston, TX, USA; c Division of Rheumatology, Department of Medicine, Perelman School of Medicine, University of Pennsylvania, Philadelphia, PA, USA.

**Keywords:** Ankylosing Spondylitis, health-related quality of life, patient-report outcomes, spondyloarthritis

## Abstract

The Patient-Reported Outcomes Measurement Information System 29-Item Health Profile (PROMIS-29) is a generic measure of health-related quality of life that is not well-studied in Ankylosing Spondylitis (AS) patients. Our objective was to investigate the reliability and validity of the PROMIS-29 in AS. About 169 consecutive AS patients were enrolled from 2017 to 2022 with 167/169 patients fully completing the PROMIS-29 in this cross-sectional study. Test–retest reliability and internal consistency was assessed using intraclass correlation coefficients (ICC) and Cronbach alpha, respectively. We studied structural validity with confirmatory factor analysis (CFA) of our hypothesized and general population models. We evaluated model fit by Chi-squared goodness-of-fit-test (χ^2^), comparative fit index, and root mean square error of approximation. A χ^2^ test was used to compare nested models. PROMIS-29 convergent validity was studied by Spearman correlation coefficients with AS-legacy measures. PROMIS-29 domains showed good test–retest reliability (intraclass correlation coefficients (ICC) > 0.7) and excellent internal consistency with Cronbach alpha > 0.9 in all subscales. CFA of only the general population model met our model fit cutoffs (χ^2^ goodness-of-fit *P*-value of 0.21, comparative fit index of 0.99, and root mean square error of approximation of 0.05). Furthermore, a nested χ^2^ test was not significantly different between our hypothesized (full) and general (reduced) model [χ^2^ (1) = 0.754, *P* > .38]. AS legacy measures showed a strong correlation (rho > |0.7|) with the extracted physical health factor. The PROMIS-29 demonstrated good reliability and construct validity in AS patients with the general population model. Further study is required to determine its clinical and research utility in AS patients.

**Key points**

- PROMIS-29 subscales are reliable in patients with AS.
- Preliminary evidence demonstrates that the underlying survey structure of the PROMIS-29 is similar in AS patients compared to the GP.
- A strong association is observed between the PROMIS-29 “Physical Health” summary score and AS-specific measures of ASDAS, BASDAI, and BASFI.

## 1. Introduction

Ankylosing Spondylitis (AS), otherwise known as radiographic axial spondyloarthritis, is a disease characterized by inflammatory back pain and radiographic disease of the axial spine with an estimated prevalence of 0.2% to 0.5% in the US population.^[1]^ AS clinical care and research have utilized traditional core measures including disease activity, pain, and physical functional limitations to assess the impact of disease.^[2]^ More recent AS-specific health-related quality of life (HRQoL) instruments are now available that better capture the full impact of disease; however, they are yet to be widely adopted in clinical care.^[3–6]^

Generic, as opposed to legacy, disease-specific patient-reported outcome measures (PROs), represent an opportunity to compare disease burden and treatment impact across different chronic conditions using a common metric. The National Institutes of Health (NIH)-funded Patient-Reported Outcomes Measurement Information System (PROMIS) incorporates both adult and pediatric PROs in physical, mental, and social health domains across a wide variety of chronic diseases and general population (GP) controls. This potentially allows investigators to compare different populations. PROMIS development, however, did not incorporate axial spondyloarthritis patients. Thus, the underlying PROMIS-29 health construct may differ in AS patients. This raises concerns thus how well the PROMIS-29 domains reflect AS HrQoL.

Hayes *et al* had found in factor analyses that the PROMIS-29 Health Profile has a 2-dimension, covarying Physical and Mental Health factor structure in the GP, generating physical and mental health summary scores.^[7]^ However, given the different physical manifestations of AS, the underlying factor structure may differ from the GP and cause inaccurate assessments of these patients’ health. Specifically, sleep disturbances are observed frequently in axial spondyloarthritis and more specifically AS.^[8,9]^ In fact, nighttime awakening in the latter half of sleep is a distinguishing feature of AS-related back pain and may lead to disrupted sleep.^[10]^ This is an impactful symptom as studies demonstrate an association between sleep and disease activity, pain, and physical functioning in AS patients.^[8]^ AS patients prioritize sleep improvement more than patients with other inflammatory disease patients.^[11]^ AS pharmacologic treatments such as Tumor Necrosis Factor inhibitors have been shown to improve sleep outcomes.^[12]^ Given the strong positive relationship between AS disease outcomes and sleep, we hypothesized the PROMIS-29 sleep domain is related to physical and emotional health, unlike the GP where it was found only to be related to emotional health in factor analysis. This potential difference in dimensionality would lead to inaccurate assessment in AS patients of PROMIS-29 physical health summary scores as currently constructed in the GP by Hayes *et al.*

The objectives of this study were to investigate the reliability and structural validity of the PROMIS-29 Health Profile in AS patients. By testing the instrument, we investigated whether the PROMIS-29 is conceptually valid and internally consistent for assessing HrQoL in AS patients.

## 2. Methods

### 2.1. Patients

Consecutive subjects were recruited from the prospective study of Ankylosing Spondylitis (PSOAS) observational cohort at UTHealth from 2017 to 2022 in a convenience sample.^[13]^ All patients at UTHealth who were seen in a rheumatology clinic that met modified New York Classification Criteria for AS, ≥18 years of age, and were fluent in English were eligible for participation.^[2]^ The research followed the Helsinki Declaration, was approved by the University of Texas Institutional Review Board (HSC-MS-07-0022), and each participating subject reviewed and signed an informed consent form.

### 2.2. Procedures

After providing written informed consent, coordinators provided paper questionnaire packets in person and/or via email. A patient subset was consecutively approached and asked to complete a second PROMIS-29 form after a 2- to 7-day washout period to assess test–retest reliability. We had previously reported our findings in PROMIS Short Forms separately.^[14]^

### 2.3. Patient-reported outcomes

Among the different formats of PROMIS, the PROMIS-29 Health Profile is a multidimensional scale. It measures 7 different domains including pain interference, physical function, anxiety, depression, fatigue, sleep disturbance [sleep], ability to participate in social roles and activities[social]), and a pain intensity numeric rating scale (NRS).^[7]^

We provided the PROMIS-29 v 2.1 distributed in paper packets for ease of use in a clinical setting. Scoring manuals for PROMIS measures (www.assessmentcenter.net/Manuals.aspx) outline development, report psychometric properties for the instrument, and describe how to identify PROMIS T scores based on raw domain summed item scores. The PROMIS-29 health domains include physical function, anxiety, depression, fatigue, sleep disturbance, ability to participate in social roles and activities, pain interference, and pain intensity NRS. Higher PROMIS scores represent more of the measured trait, so the interpretation of directionality varied if the domain was a positive trait (higher scores better) versus a symptom (higher scores indicate more severe symptoms).

In the PSOAS cohort, patients report AS-disease-specific PROs, including the following instruments used in AS clinical care: Ankylosing Spondylitis Disease Activity Score (ASDAS),^[15]^ Bath Ankylosing Spondylitis Disease Activity Index (BASDAI),^[16]^ Bath Ankylosing Spondylitis Functional Index (BASFI),^[17]^ Physician Global Assessment of Disease Activity (PhGADA),^[18]^ Pain Numeric Rating Scale (Pain NRS), and Patient Global Assessment of Disease Activity (Global NRS).^[2]^

### 2.4. Covariates

We obtained sociodemographic information including age, gender, race/ethnicity, education, smoking, comorbidities, work status, and AS duration. Medication use, comorbidities, and serum inflammatory markers (e.g. C-reactive protein [CRP], erythrocyte sedimentation rate) were also recorded at each visit in addition to axial skeleton radiographs every 2 years.

### 2.5. Statistical analysis

Central tendency and distribution were calculated by mean (SD) or median (IQR) for continuous normal versus nonnormal data, respectively. Frequencies and percentages were descriptively reported for categorical variables. We studied reliability through test–retest reliability (in the subset completing the retest packet) and internal consistency (all patients) of the PROMIS-29 domains/subscales using intraclass correlation coefficients (ICC) and Cronbach alpha, respectively, with thresholds of ≥0.7 considered acceptable.^[19]^ We also reported standard error of measurement and the minimal detectable change for each PROMIS-29 domain/subscale.^[20]^

**Figure 1.**
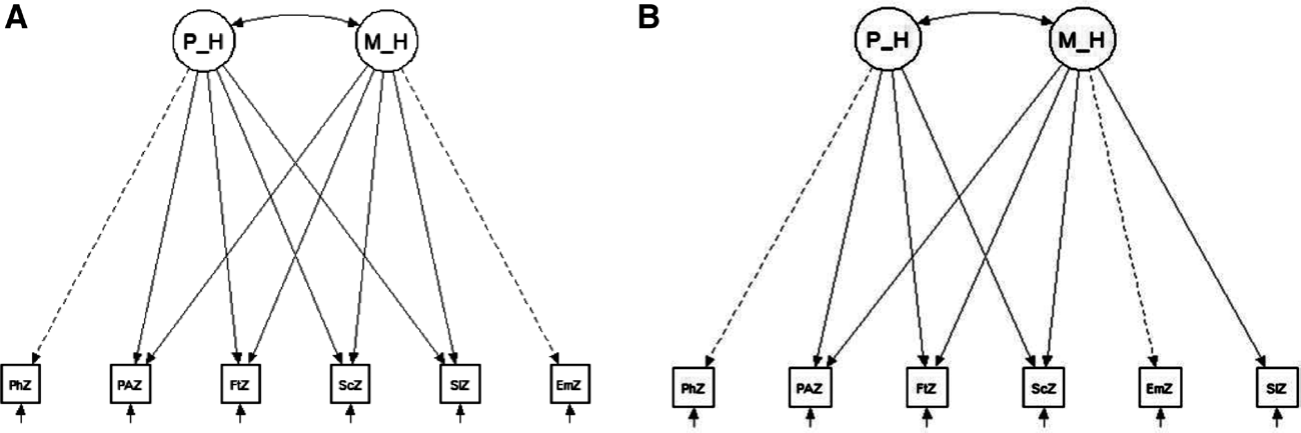
Proposed AS and general populations dimensionality of the PROMIS-29. Proposed models of (A) hypothesized model (additional relationship between the sleep domain and Physical Factor) and (B) general population model. Circles represent latent constructs (factors). Squares represent observed/measured variables—PROMIS short forms. Observed variables are connected to latent factors by straight arrows, which represent the loading (variance in the observed variable explained by the latent factor). Double-headed arrows represent covariance between latent factors. The dotted lines represent the strongest/defining correlations of latent factors. Short arrows represent error terms, P_H = Physical Health Factor, M_H = Mental Health Factor, PhZ = Physical Function, PAZ = Pain, FtZ = Fatigue, ScZ = Ability to Participate in Social Activities [Social], SlZ = Sleep Disturbance [Sleep], EmZ = Emotional Distress.

We studied structural validity, or the degree to which the scores of a health-related PRO reflect the dimensionality of the construct measured^[21]^ by conducting confirmatory factor analyses (CFA) using the underlying 2-factor, (physical and mental health) general population (GP) model as described by Hays *et al* (3) (Fig. 1B). We also conducted CFA with our alternative, hypothesized AS model that showed a disease-specific relationship between sleep disturbances and the physical health factor (Fig. 1A). We reported PROMIS Z-scores derived from the calculated T scores as described by Spritzer and Hays (http://www.healthmeasures.net/media/kunena/attachments/257/PROMIS29_Scoring_08082018.pdf) for all PROMIS-29 domains. A combined pain domain was averaged from pain intensity and pain interference T scores. Similarly, the emotional distress domain was averaged from depression and anxiety T scores. The practical fit of the model was evaluated using the Chi-squared goodness-of-fit-test, comparative fit index (CFI), and the root mean square error of approximation (RMSEA). Good model fit was defined by a Chi-square goodness-of-fit ≥ 0.5, CFI > 0.95, and RMSEA < 0.06. Since the models are nested, a Chi-square difference test was conducted to compare the fit of the hypothesized and GP model.^[22]^

We used the Spearman correlation coefficient to determine the correlation between the factor summary scores and legacy measures. A rho > |0.7| was considered strong correlation. Given the directionality of PROMIS and legacy measures, we hypothesized the physical and mental health summary scores would have a strong, inverse correlation with ASDAS, BASDAI, BASFI, Pain NRS, and Global NRS. PROs with incomplete data were excluded from their respective analyses. Analyses were done in IBM SPSS version 24 and R version 4.01 using the lavaan package (http://lavaan.ugent.be/).

## 3. Results

### 3.1. Patient characteristics

A total of 169 patients were enrolled and completed the surveys between September 2017 and January 2022. Twenty 4 of the 88 patients (27.3%) from May 2018 through November 2018 completed the retest packet. This sample included a diverse spectrum of AS characteristics (Table 1). Patients were mostly male (69%) and White (81%) with a mean (SD) age of 51 (±15) years. The mean symptom duration was 25 (±13) years. In those who had available CRP lab values (135/169, 73%), over half (53%) had high or very high disease activity by ASDAS. We had 167/169 patients with complete PROMIS-29 data.

**Table 1:** Participant characteristics (*N* = 169).

Characteristic	*N*	Value (%)
Age (Mean ± SD; years)	169	50.85 ± 14.77
Male gender	169	82 (69%)
Race	169	
White		96 (81%)
Other		23 (19%)
Education	169	
High school		17 (13%)
College		110 (87%)
Employment status	169	
Full time		84 (66%)
Not Working		32 (25%)
Disabled		11 (9%)
Body mass (Mean ± SD; kg)	169	87.31 ± 20.40
Height (Mean ± SD; cm)	169	171.31 ± 10.21
AS symptom duration (Mean ± SD; years)	169	25.47 ± 13.32
ASDAS*	135	
Inactive		27 (27%)
Low		34 (35%)
High–very high		72 (53%)
BASDAI	165	
≥4		83 (51%)
<4		82 (49%)
Biologic DMARD usage†	162	76 (47%)

* Missing = 34 due to lack of BASDAI score and/or CRP labs.

† Missing = 7 due to incomplete medication list.

### 3.2. PROMIS-29 reliable in AS

All PROMIS-29 Health Profile domains showed excellent internal consistency with Cronbach alpha ranging from 0.86 to 0.98 (Table 2) in our AS patient sample. Test–retest intraclass correlation coefficients ranged from 0.79 (physical function) to 0.94 (fatigue).

**Table 2:** PROMIS measure scores in AS patients.

	*N*	Mean	Median	Std. Deviation	Range	Minimum	Maximum	Test–retest ICC (95% CI)*	Cronbach alpha (95% CI)	Standard error of measurement	Minimal detectable change
PROMIS-29 Subscale											
Physical function	168	46.63	45.30	8.67	30.00	26.90	56.90	.79 (.52, .90)	.91 (.88, .93)	3.97	11.01
Depression	167	46.11	41.00	7.33	28.40	41.00	69.40	.89 (.77, .95)	.89 (.86, .92)	2.43	6.73
Anxiety	168	47.83	40.30	9.30	41.30	40.30	81.60	.89 (.77, .95)	.92 (.90, .94)	3.08	8.54
Fatigue	168	50.55	48.60	10.66	42.10	33.70	75.80	.94 (.88, .97)	.95 (.93, .96)	2.61	7.23
Sleep	169	51.53	52.40	8.53	41.30	32.00	73.30	.90 (.79, .95)	.86 (.81, .89)	2.69	7.47
Social roles and activities	169	37.41	37.30	8.86	36.70	27.50	64.20	.93 (.85, .97)	.96 (.95, .97)	2.34	6.49
Pain interference	169	52.73	53.90	10.06	34.00	41.60	75.60	.84 (.63, .93)	.97 (.96, .98)	4.02	11.15
Pain intensity [0–10 NRS]	168	3.26	2.5	2.58	10.00	0	9	.86 (.73, .95)	xxx	0.96	2.67

* *n* = 24 in this subset.

### 3.3. General population model has better fit and parsimony compared to hypothesized AS model

To determine the best dimensionality of PROMIS-29 domains in AS patients forming physical and mental health factors we performed CFA of our hypothesized AS and GP model (Fig. 1A and B, respectively). From a model fit perspective, our hypothesized factor structure/model had a Chi-squared test *P*-value of 0.07, a CFI of 0.98, and RMSEA of 0.09, not meeting all predefined cutoffs for good model fit. The GP factor structure/model (Chi-squared test *P*-value of 0.21, a CFI of 0.99, and RMSEA of 0.05) was similar; however, it met our predefined model fit cutoffs for good fit. A 0.26 and 0.24 covariance, demonstrating moderate relationships between the extracted physical and mental health factors, were present in the hypothesized and GP models, respectively. A nested Chi-square difference test comparing the 2 models showed no significant difference between our hypothesized and GP model [χ^2^ (1) = 0.754, *P* > .38].

### 3.4. Extracted factors show correlation with AS-specific measures

To study the convergent validity of the PROMIS-29, we studied the correlation of our AS-specific measures with the extracted factors. First, we obtained “Physical Health” and “Mental Health” summary scores by using the PROMIS-29 domain scores and factors loadings (relationship between the PROMIS-29 domains and factors) from the general population model CFA (Supplementary Table S1, http://links.lww.com/MD/L772) for each patient. We then compared these summary scores to legacy measures of ASDAS, BASDAI, BASFI, CRP, Global NRS, Pain NRS, and PhGADA. The physical health summary scores (higher representing greater physical health) showed a strong correlation (rho > |0.7|) with ASDAS, BASDAI, BASFI, Pain NRS, and Global NRS (Table 3). The Mental Health summary score (higher scores representing greater emotional distress), showed a strong correlation with ASDAS, BASDAI, and Global NRS but only moderate with Pain NRS or BASFI. A moderate correlation was observed between PhGADA with the physical health compared to a weak correlation observed between PhGADA and the mental health. C-reactive protein had weak correlation with both summary scores.

**Table 3:** Correlation of legacy measures with PROMIS-29 physical and mental health summary scores†

	Physical health	Mental health
BASDAI	−.79*	−.75*
BASFI	−.80*	−.66*
ASDAS	−.74*	−.71*
CRP	−.32*	−.29*
Pain NRS	−.70*	−.65*
Global NRS	−.84*	−.78*
Physician VAS	−.43*	−.28*

* Correlation is significant at the 0.01 level (2-tailed).

† Summary scores derived from factor loadings of observed PROMIS-29 domains with extracted factors from confirmatory factor analysis to have a single Physical Health and Mental Health score per patient. See Supplementary Table S1 for further details regarding factor loadings.

## 4. Discussion

To the best of our knowledge, this study is the first to examine the structural validity of the PROMIS health profile instruments in AS patients to help determine their clinical and research use in this patient population. In our study, the PROMIS-29 showed good reliability, structural validity with the GP model, and convergent validity with AS-legacy measures. Alternative, disease-specific models demonstrated a worse fit based on predefined model fit cutoffs when we studied dimensionality/structural validity. Furthermore, when testing the chi-square difference, we were unable to reject the null hypothesis that there was no difference between these 2 models tested. These findings may suggest the more parsimonious *GP* model should be used in AS patients. Simply stated, the PROMIS-29 measures reflect health-related quality of life in AS patients like the GP and can be interpreted similarly.

Our study adds to understanding the applicability of PROMIS-29 in AS patients, supporting the use of the current PROMIS-29 instrument as opposed to alternate models. The PROMIS-29 has been studied in other rheumatic diseases as an HrQoL instrument; however, studies to date have focused on the performance of the 7 domains independently.^[23–26]^ Given the complexity of HrQoL in patients with rheumatic diseases, it was important to examine the dimensionalities of summary scores specifically in AS patients. By adapting the factor summary scores in AS patients through sample-specific factor loadings, we also showed AS-specific measures are strongly related to physical health, similar to other HrQOL measures.^[27]^

We selected the PROMIS-29 health profile and a priori suggested that AS patients’ factor structure compared to the GP would be different due to their rheumatic disease-specific characteristics. However, our hypothesized relationship between the sleep domain and the “physical health” factor did not show a stronger fit compared to the GP model (Fig. 1A and B). We suspect that this may be explained by the fact that sleep disturbances are multifactorial and probably many of these concepts are interrelated in AS, similar to the GP. Particularly the covariance relationship between the physical health and mental health factors/summary scores may be accounting for sleep disturbance in both domains.

Strengths of this study included the use of a well-characterized AS cohort of US patients with regularly collected AS-specific measures. All patients met modified New York Criteria for AS, creating a homogenous patient sample. We also evaluated the performance of PROMIS measures within the context of usual care.

Our study has limitations. AS/AxSpA-specific HrQoL instruments were not available for comparison. Our sample size may not have been enough to detect small differences in factor loading. Similarly, we studied test–retest reliability in a consecutive but small portion of our patient sample although these did meet our predefined thresholds. The highly educated, English-speaking-only, largely Caucasian demographics of our tertiary referral, SpA program patient sample may impact generalizability. Furthermore, by only including patients who met modified New York Criteria for AS we excluded patients with nonradiographic axial spondyloarthritis (nr-AxSpA) and others in the disease spectrum. Our study thus can only be taken in an indirect context to nr-AxSpA patient populations.

In conclusion, this study demonstrates preliminary evidence of the reliability and construct validity of the PROMIS-29 Health Profile in AS patients. We showed that the PROMIS-29 in AS patients has similar dimensionality (structural validity) to the general adult population. Furthermore, convergent validity was demonstrated with the physical health factor demonstrating strong correlation with legacy measures similar to other generic HrQOL measures such as the SF-36. The potential implications of these findings suggest that the PROMIS-29 survey may be a generic HrQOL measure that reflects AS patient health. Further study is required to determine if the 2-factor Hays model found in our patient population can be reproduced in independent samples of AS patients or if it demonstrates a different, disease-specific structure. Future work that examines the convergent validity of the PROMIS-29 summary scores with disease-specific HrQOL instruments and discrimination of the PROMIS-29 in treatment initiation scenarios will help to further elucidate how the PROMIS-29 can be used in clinical contexts.

## Acknowledgments

The authors are grateful to the patients and their families for their participation and support of this study. We also thank the PSOAS investigators and Center for Clinical and Translational Sciences at UTHealth. Special thanks to Dr Zsuzsanna McMahan at UTHealth Houston for manuscript editing.

## Author contributions

**Conceptualization:** Mark C. Hwang, Cynthia Bell, Alexis Ogdie, John Reveille.

**Data curation:** Mark C. Hwang, Cynthia Bell, Yvette Farran, Charles Green, John Reveille.

**Formal analysis:** Mark C. Hwang, Cynthia Bell, Alexis Ogdie, Charles Green, John Reveille.

**Funding acquisition:** Mark C. Hwang, John Reveille.

**Investigation:** Mark C. Hwang, Charles Green, John Reveille.

**Methodology:** Mark C. Hwang, Cynthia Bell, Alexis Ogdie, Charles Green, John Reveille.

**Project administration:** Mark C. Hwang.

**Resources:** Mark C. Hwang, Yvette Farran.

**Software:** Mark C. Hwang.

**Supervision:** Mark C. Hwang, Cynthia Bell, Charles Green, John Reveille.

**Validation:** Mark C. Hwang, John Reveille.

**Visualization:** Mark C. Hwang.

**Writing—original draft:** Mark C. Hwang.

**Writing—review & editing:** Mark C. Hwang, Cynthia Bell, Yvette Farran, Alexis Ogdie, Charles Green, John Reveille.

## Supplementary Material


