# Supplementary material for: Reliability and validity of the PROMIS-29 health profile in Ankylosing Spondylitis patients: A cross-sectional study
Source: Medicine (Baltimore). 2024 Mar 1;103(9):e37251. doi: 10.1097/MD.0000000000037251 (PMC10906602; doi:10.1097/MD.0000000000037251)
Supplement: Supplementary file 1 [file medi-103-e37251-s001.docx]

| **Supplemental Table 1. Standard Factor Loadings from General Population Confirmatory Factor Analysis** | | |
| --- | --- | --- |
|  | **Physical Health Factor** | **Mental Health**  **Factor** |
| Physical Function | 1.00^*^ | 0.00 |
| Pain | -0.542^*^ | -0.860^*^ |
| Fatigue | -0.489^*^ | -0.796^*^ |
| Social | 0.745^*^ | 0.838* |
| Emotional Distress | 0.00 | -1.00^*^ |
| Sleep | 0.00 | -0.687 |
| * Correlation is significant at the 0.05 level (2-tailed). | | |
|  | | |
